# Dietary Supplement Enriched in Antioxidants and Omega-3 Promotes Glutamine Synthesis in Müller Cells: A Key Process against Oxidative Stress in Retina

**DOI:** 10.3390/nu13093216

**Published:** 2021-09-16

**Authors:** Maryvonne Ardourel, Chloé Felgerolle, Arnaud Pâris, Niyazi Acar, Khaoula Ramchani Ben Othman, Natsuko Ueda, Rafaelle Rossignol, Audrey Bazinet, Betty Hébert, Sylvain Briault, Isabelle Ranchon-Cole, Olivier Perche

**Affiliations:** 1 UMR7355, Experimental and Molecular Immunology and Neurogenetics, CNRS Orléans Campus, 3B rue de la Ferollerie, 45100 Orléans, France; maryvonne.ardourel@univ-orleans.fr (M.A.); chloe.felgerolle@pasteur.fr (C.F.); arnaud.paris@univ-orleans.fr (A.P.); natsuko.ueda@enscm.fr (N.U.); rafaelle.rossignol@laposte.net (R.R.); bazinet.audrey@live.fr (A.B.); hebert.betty@neuf.fr (B.H.); sylvain.briault@chr-orleans.fr (S.B.); 2 Experimental and Molecular Immunology and Neurogenetics, University of Orléans, 45100 Orléans, France; 3 Eye and Nutrition Research Group, Centre des Sciences du Goût et de l’Alimentation, AgroSup Dijon, CNRS, INRAE, Université Bourgogne Franche-Comté, 21000 Dijon, France; niyazi.acar@inrae.fr; 4 Laboratory of Sensorial Biophysical, University of Clermont, 63000 Clermont-Ferrand, France; rbo.khaoula@live.fr (K.R.B.O.); isabelle.ranchon-cole@uca.fr (I.R.-C.); 5 Genetic Department, Regional Hospital, 45100 Orléans, France

**Keywords:** nutritional supplementation, Müller cells, glutamate, glutamine, oxidative stress

## Abstract

To prevent ocular pathologies, new generation of dietary supplements have been commercially available. They consist of nutritional supplement mixing components known to provide antioxidative properties, such as unsaturated fatty acid, resveratrol or flavonoids. However, to date, only one preclinical study has evaluated the impact of a mixture mainly composed of those components (Nutrof Total^®^) on the retina and demonstrated that in vivo supplementation prevents the retina from structural and functional injuries induced by light. Considering the crucial role played by the glial Müller cells in the retina, particularly to regulate the glutamate cycle to prevent damage in oxidative stress conditions, we questioned the impact of this ocular supplement on the glutamate metabolic cycle. To this end, various molecular aspects associated with the glutamate/glutamine metabolism cycle in Müller cells were investigated on primary Müller cells cultures incubated, or not, with the commercially mix supplement before being subjected, or not, to oxidative conditions. Our results demonstrated that in vitro supplementation provides guidance of the glutamate/glutamine cycle in favor of glutamine synthesis. These results suggest that glutamine synthesis is a crucial cellular process of retinal protection against oxidative damages and could be a key step in the previous in vivo beneficial results provided by the dietary supplementation.

## 1. Introduction

Müller cells play a crucial role in maintaining the complex architecture and function of the retina. They are considered as the principal glial cells of the retina since they perform many of the functions carried out by astrocytes, oligodendrocytes and ependymal cells in other regions of the central nervous system [1]. Müller cells are radially oriented, spanning the entire retina from the inner to the distal end of the outer nuclear layer (ONL) and presenting close contacts with photoreceptors and bipolar cells. These strong interactions with retinal neurons ensure numerous metabolic exchanges maintaining retinal integrity, in particular by participating in the control of retinal homeostasis and especially redox and glutamatergic homeostasis [2,3,4,5].

Indeed, in the tripartite glutamatergic synapse (photoreceptor, bipolar and Müller cells), Müller cells regulate the glutamate/glutamine metabolic cycle in order to control glutamate level. This cycle aims to recapture glutamate from the synaptic cleft, thus avoiding excitotoxicity [6,7] and providing glutamine to replenish neurotransmitter pools in neurons. Glutamate internalization into Müller cells is supported by high-affinity Na+-dependent transporters (EAAT1 or GLAST). In Müller cells, glutamate is partly amidated by glutamine synthetase (GS) into glutamine [8,9], which is exported to the extracellular compartment via the Na+-dependent transporter, SN1. Glutamine is taken into photoreceptor cells through a Na+-dependent transporter (SNAT) and converted into glutamate by glutaminase [6].

Interestingly, when Müller cells are exposed to oxidative stress conditions, the cycle is deviated and glutamate is used to synthesize glutathione, a crucial antioxidant molecule that prevents neuronal damage by neutralizing Reactive Oxygen Species (ROS) [10,11]. However, when ROS production is too important, the detoxification machinery is outdated, leading to a deleterious increase of extracellular glutamate concentration due to imbalanced glutamate/glutamine metabolic cycle [6]. This mechanism, known as the glutamate excitotoxicity, is an important contributor to ocular conditions ranging from glaucoma to Age-Related Macular Degeneration (AMD) [12,13]. As a consequence, glutamate, which is the dominant excitatory neurotransmitter in the mammalian central nervous system (CNS) including the retina [6], needs a tightly control of its level in physiological or oxidative stress conditions to prevent any excitotoxicity phenomenon. Therefore, the importance of Müller cell functions makes these cells suitable targets for prevention of numerous retinal pathologies [14].

To prevent ocular pathologies, clinical trials conducted by the Age-Related Eye Disease Study group (AREDS) demonstrated the efficiency of nutritional treatment such as single supplementation in vitamin C, vitamin E or beta-carotene [15]. Since, to improve this clinical therapeutic effect, new generations of nutritional supplements have been developed as a mixture containing either vitamin C, vitamin E, beta-carotene or other components such as fatty acid, resveratrol or flavonoids, based on their anti-oxidative properties. However, limited research has been conducted with these complex formulations, limiting our understanding on their cellular or molecular mechanism on retinal protection. To our knowledge, only one preclinical study using one of the most recommended mixture in clinic was conducted [16]. In this work, the authors demonstrated in vivo in rats that nutritional supplementation had no impact on basal retinal function assessed by electroretinography, nor on retinal structure [16]. More interestingly, they evidenced that it protected the retina from light-induced retinal degeneration known to have a predominant oxidative stress process. Therefore, it was suggested that this formulation exerts anti-oxidative properties in the retina in vivo.

Consequently, considering the importance of Müller cell function in retinal anti-oxidative mechanisms, we investigated the impact of this same formulation on the glutamate/glutamine metabolic cycle with or without sub-lethal oxidative stress conditions. In our experimental conditions, we demonstrated in vitro that the formulation modifies the glutamate/glutamine metabolic cycle in favor of glutamine synthesis. Whereas supplementation did not affect glutamate uptake, it increased glutamine release in extracellular medium suggesting that supplemented Müller cells deviated their intracellular metabolic pathway to ensure a high level of glutamine synthesis. Therefore, glutamine synthesis appears to be a pivotal mechanism involved in retinal protection.

## 2. Materials and Methods

### 2.1. Animals

Retinal cell cultures were obtained using retinas of C57BL/6J mice. Animals were maintained under 22 °C and 55% humidity with a 12 h:12 h dim light–dark cycle (25 lux, light ON at 7 a.m.). All animal experimental protocols were reviewed by the ‘‘Ethics Committee for Animal Experimentation of CNRS Campus Orleans’’ (CCO N_3) and approved by the French National Committee of Ethical Reflexion for Animal Experimentation, under N_ CLE CCO 1100.

### 2.2. Müller Cells Culture

Müller cells were isolated from retinas between postnatal day P5 to P10 using a protocol modified from Hicks and Courtois [17]. Briefly, following euthanasia, both eyes were rapidly enucleated and immersed in culture medium, i.e., Dulbecco’s modified Eagle’s medium high glucose (DMEM, Thermofisher, France) containing 10 units/mL penicillin and 0.1 mg/mL streptomycin (Thermofisher, France), 20% FBS (Thermofisher, France) and 2 mM glutamine (Thermofisher, France), overnight in the dark at 4 °C. Eyes were subsequently transferred into DMEM containing 10 units/mL penicillin, 0.1 mg/mL streptomycin, and 2 mM L-glutamine provided with 0.1% trypsin/EDTA (Thermofisher, France) and 70 U/mL of collagenase (Thermofisher, France) at 37 °C for 60 min. Retinas were then carefully dissected from the other ocular tissues and dissociated by trituration into culture medium. The resulting cell suspension was then seeded into 24-well plates (6 wells per mice) containing culture medium at 37 °C, 5% CO_2_, hygrometry 95%. Three days later, culture plates were shaken vigorously to detach non-adherent cells, which were removed by aspiration before adding fresh culture medium. Then, medium was changed every 2 days. The culture was composed of > 98% Müller cells as evidenced by immunopositivity for glutamine synthetase (GS), glutamate aspartate transporter (GLAST) as well as immunonegativity for NeuN (neurons) (Data not shown).

### 2.3. Treatment and Experimental Conditions

**Figure 1:**
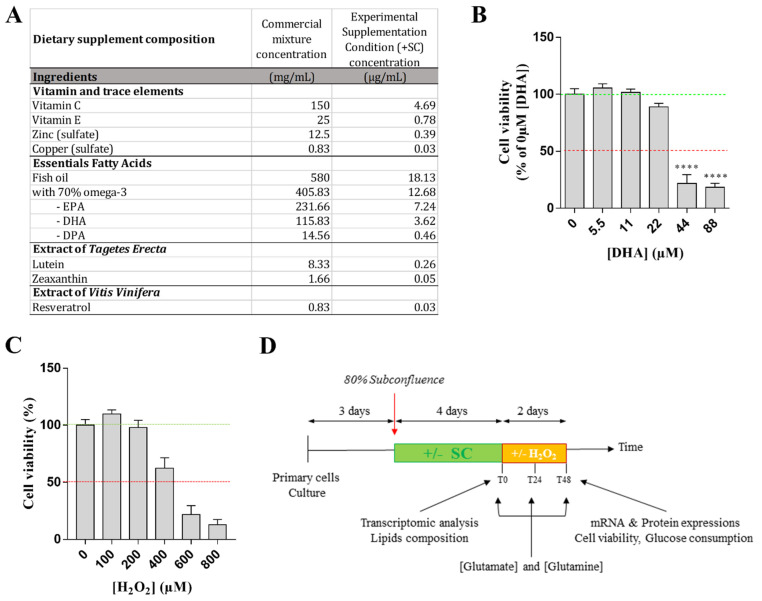
Dietary supplement composition and in vitro experimental design. (**A**) Dietary supplement composition. (**B**) Effect of dietary supplementation, expressed as equivalent DHA (µM), on Müller cells viability. All results are presented as mean ± SEM. Significant differences between groups are noted by **** *p* < 0.0001. (**C**) In vitro experimental design. SC means “supplementation condition”. (**D**) Experimental design of supplementation and oxidative stress treatment.

Supplementation treatment: The supplement is a commercially available supplement (Nutrof Total^®^) whose composition is detailed in Figure 1A. It was extemporaneously prepared at 1.1 mM equivalent DHA (Docosahexaenoic Acid, main omega-3 essential fatty acids) in DMSO 100% before dilution to a final working solution at 11 µM equivalent DHA and 0.1% DMSO in culture medium. The final DMSO concentration was inferior to 0.1%. This Equivalent DHA concentration was chosen in accordance with the literature (personal communication Ramchani-Ben Othman, K) [16]. In addition, we had shown that at this concentration the supplement had no effect on Müller cell viability (Figure 1B). Cells were incubated for 4 days with culture media containing supplementation at 11 µM equivalent DHA (+SC, meaning “with Supplementation Condition”) or no supplementation (−SC, meaning “without Supplementation Condition”).

Sub-lethal oxidative stress experimental conditions: Sub-lethal oxidative stress was induced with H_2_O_2_ at 100 or 200 µM in DMEM without glutamine nor red phenol but containing 10 units/mL penicillin and 0.1 mg/mL streptomycin. These concentrations were chosen since they did not affect cell viability or glucose consumption (Figure 1C).

Glutamate experimental conditions: High glutamate concentration (1 mM) in culture medium with nor glutamine, nor red phenol, or FBS was used for metabolic test. This concentration was chosen [18,19] in order to mimic glutamate toxicity involved in retinal degeneration conditions [20,21].

### 2.4. Experimental Design

After 3 days of culture corresponding to 80% confluence, cells were incubated with (+SC) or without (−SC) dietary supplementation. Four days later (T0) media was replaced with fresh culture media containing 1mM of glutamate and cells were untreated (vehicle) or treated with 100 µM H_2_O_2_ or with 200 µM H_2_O_2_. The different assays were performed at T0, T24 (+24 h) and T48 (+24 h) (Figure 1D).

Extra- and intra-cellular glutamate and glutamine assays as well as glucose consumption experiments were performed at T0, T24 and T48. mRNA expression, protein expression and cell viability were performed at T48. Lipid composition and transcriptomic profiles were evaluated at T0 (Figure 1C). Total amount of proteins was determined for each well and used to normalize results.

Each experiment was performed at least 5 times and had been carried out by 3 different experimenters.

### 2.5. Glutamate and Glutamine Dosages

Glutamate and glutamine concentrations were determined by modified GLN1 Kit (Sigma, Saint-Quentin-Fallavier, France). Glutamate concentration was directly evaluated in the sample by dehydrogenation of the L-glutamate to α-ketoglutarate accompanied by reduction of NAD+ to NADH. The conversion of NAD+ to NADH was measured spectrophotometrically and was proportional to the amount of oxidized glutamate. Determination of L-glutamine was a two-step reaction: (A) deamination of L-glutamine into L-glutamate by glutaminase and (B) dehydrogenation of the L-glutamate to α-ketoglutarate by glutamate dehydrogenase. Hence, the amount of glutamine converted into glutamate in the samples was obtained by the difference between glutamate levels without and with glutaminase. Therefore, two mixes were prepared: sodium acetate buffer (0.4 M, pH 4.8) with 25% of glutaminase (from Escherichia coli, Sigma, Saint-Quentin-Fallavier, France) and the same sodium acetate buffer without glutaminase. To determine the extracellular glutamate and glutamine concentrations, 90 µL of assessed medium were mixed to 60 µL of each mix. After incubation at 37 °C for 1 h, glutamate concentration was dosed in L-GLDH buffer composed of Tris-EDTA-hydrazine buffer (0.33 M Tris-HCl pH 9.0, 1.33 mM EDTA, 2% hydrazine pH 9.0) with 2 mM β-NAD+, and 6.67 mM ADP, in UV microplates. Thirty percent L-GLDH (L-glutamic dehydrogenase from bovine liver, Sigma, Saint-Quentin-Fallavier, France) enzyme in H_2_O was added to samples, and plates were left for 1 h in the dark at room temperature. Absorbances at 340 nm were then measured (CLARIOstar imager, BMG LABTECH, Ortenberg, Germany), and values recorded before the addition of L-GLDH was subtracted. Glutamate concentration was reported to sample protein concentration. Results were expressed as percent of medium glutamate concentration used for the stimulation. In addition, Aera Under the Curve (AUC) over the 48 h of test were calculated (Arbitrary Unit—AU) using MicroCal software. For intracellular glutamate and glutamine concentrations, cell suspensions were centrifuged for 30 min, 13,000× *g*, at 4 °C, and cellular pellets were homogenized in sodium acetate buffer (0.05 M, pH 5.0) with sonification. Supernatants were collected and underwent the standard dosage as described previously.

### 2.6. Cell Viability Test

Cell viability was evaluated using MTT colorimetric assays as described previously [22]. Briefly, medium was removed, 500 µL of RPMI medium containing MTT (0.5 mg/mL) were added to each well and incubated for 4 h at 37 °C. After incubation, 50 µL of SDS solution (10% of SDS in DMSO added with pure acetic acid 0.6% final) were added to each well to solubilize the formazan product. The plate was left in the dark overnight at room temperature. Absorbance of each well at 610nm was measured using CLARIOstar imager (BMG LABTECH, Ortenberg, Germany). Results were expressed as percent of unsupplemented and untreated cells.

### 2.7. Glucose Consumption

Glucose concentration in culture media was determined by spectrophotometric methods using the Glucose GOD-PAD kit (Prolabo, Paris, France) according to the manufacturer’s instructions. Absorbance at 500 nm was measured for each well using the CLARIOstar imager (BMG LABTECH, Ortenberg, Germany). Glucose consumption was calculated as the difference between the glucose concentration at T48 and the initial glucose concentration at T0 (1 g/L). Results were expressed as percent of untreated cells.

### 2.8. Western Blotting

Proteins were extracted from Müller cells culture. Briefly, Müller cells were homogenized in RIPA buffer (10 mM Tris-HCl, pH 7.6; 1 mM EDTA; NaCl 0.15 mM; Igepal 1%; SDS 0.2%; supplemented with protease cocktail inhibitors (Pierce, Paris, France), centrifugated for 30 min at 13,000× *g* and at 4 °C. Supernatants were collected. Protein concentration was determined in supernatants by BCA protein assay kit (Pierce, Paris, France). Fifteen µg of protein were run on SDS/PAGE gels (4–10%, *w*/*v*), transferred to a nitrocellulose membrane, and probed with primary antibodies from Sigma-Aldrich (Illkirch, France) (anti-GS, 1:1000; anti-GFAP, 1:1000) or from AbCam (Paris, France) (anti-EAAT1, 1:500; anti-GLS, 1:500; anti-SN1, 1:500) then secondary HRP-antibody (1:4000) (Promega, Paris, France) and Sigma-Aldrich (Illkirch, France). Mouse anti-beta Actin antibody (1:4000) (AbCam, Paris, France) was used for loading control. Immunoreactive bands were quantified using PixI software (Ozyme, Paris, France). Western blots were performed three times. Representative pictures are shown in figures. Results are presented as percent of untreated cells.

### 2.9. Quantitative RT-PCR

Quantitative RT-PCR was performed using Taqman technology (Applied technologies, Cleveland, OH, USA) as described previously [23]. Briefly, total RNA was isolated from Müller cells culture using TRIzol reagent (Ambion, Life Technologies, Carlsbad, CA, USA), quantified and reverse transcripted (Superscript iii reverse transcriptase, Invitrogen, Life Technologies). Real-time PCR reactions were performed in the Mx3005P Agilent (Applied, Life Technologies) with fivefold dilution of cDNA, 200 nM of each Taqman primer using the Expression Master Mix (Applied, Life Technologies). Data were normalized to the reference standard RNA 18S, analyzed by ΔΔCt method and expressed as LogFC (Fold Change) compared to basal condition (−SC). Each measurement was performed three times.

### 2.10. Expression Arrays

Total RNA was extracted using TRIzol reagent (Invitrogen, Waltham, MA, USA) according to the manufacturer’s instruction. Genome-wide transcriptional profiling was performed by Mice Exon 1.0 ST arrays (Affymetrix, Santa Clara, CA, USA) following manufacturer’s instructions and as described previously [24,25]. Each condition was performed in duplicate. GeneChip CEL files were analyzed using GeneSpring (Agilent Technologies, Santa Clara, CA, USA). The log2 values were obtained and the microarray data were normalized using RMA16 algorithm. Statistical data analyses were done on all samples for each group. Similarly, enrichment rankings were based on all samples per group. Data were filtered with GeneSpring analysis using a cutoff of at least 1.5 times up or down (Fold Change 1.5, FC1.5). Subsequently, one-way ANOVA was performed on a filtered list. The Benjamini–Hochberg false discovery rate (FDR) was set to 5%, and the P value was set to <0.05 (ANOVA test). The Database for Annotation, Visualization, and Integrated Discovery (DAVID) was used to find gene ontology-enriched terms. Data were confirmed by quantitative RT-PCR. Quantitative RT-PCR were performed using Taqman technology (Applied Technologies) as described previously [25]. Data were analyzed by ΔΔCt method and normalized to the reference standard RNA 18S. Each measurement was performed three times.

### 2.11. Fatty Acid Composition

Müller cells were homogenized in PBS-1X after scraping, centrifuged at 10,000× *g* for 10 min at 4 °C and conserved at −80 °C until lipid extraction.

Total lipids extraction: Total lipids were extracted from cell homogenates following Folch’s method [26].

Determination of lipid class distribution: The distribution of lipids into phospholipids, triglycerides, free fatty acids, free cholesterol, and cholesteryl esters in cells was determined using a combination of thin-layer chromatography on silica gel-coated quartz rods and flame ionization detection (Iatroscan^®^ system, Iatron, Tokyo, Japan), according to Ackman’s technique [27] (Ackman, 1981). The values obtained for each compound were corrected according to their response factor using specific calibration curves, as published previously [28]. Data were reported as a percentage of the total lipids in the sample.

Determination of fatty acid composition: Lipids were transmethylated using boron trifluoride in methanol according to Morrison and Smith (1964) [29]. The fatty acid methyl esters formed by transmethylation were analyzed on a Trace 1310 gas chromatograph (Thermofisher, Waltham, MA, USA) fitted with a CPSIL-88 column (100 m × 0.25 mm i.d., film thickness 0.20 µm; Varian, Les Ulis, France). Hydrogen was used as a carrier gas (inlet pressure 210 kPa). The oven temperature was held at 60 °C for 5 min, increased to 165 °C at 15 °C/min and held for 1min, then increased to 225 °C at 2 °C/min, and finally held at 225 °C for 7 min. The injector and the flame ionization detector were maintained at 250 °C. Fatty acid methyl esters were identified by comparison with commercial and synthetic standards. The data were computed using the EZChrom software (Agilent Technologies, Massy, France) and reported as a percentage of the total fatty acids.

### 2.12. Statistical Analysis

All results are expressed as mean ± SEM. Data analysis was performed using GraphPad Prism version 8.0.0 for Windows, GraphPad Software, San Diego, California, USA. For supplementation effect, we performed Mann–Whitney’s non-parametric test, whereas the oxidative stress effect was investigated using a one-way ANOVA followed by a *post-hoc* Holm–Sidak test. Statistical significance was defined as *p* < 0.05. Significant differences between groups are noted by * *p* < 0.05; ** *p* < 0.01; *** *p* < 0.001; **** *p* < 0.0001.

## 3. Results

### 3.1. Effects of Nutritional Supplementation on Müller Cells Basal Conditions

**Figure 2:**
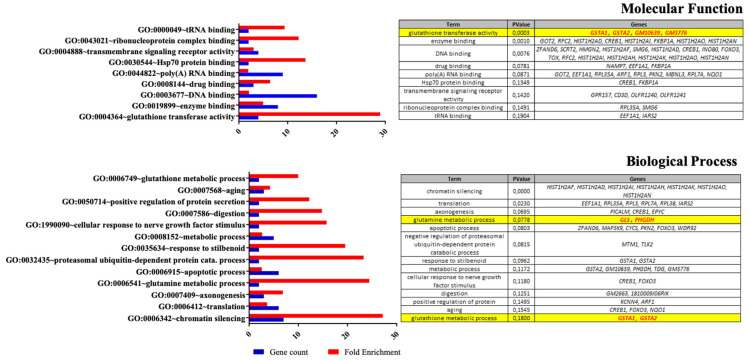
Transcriptomic profile in Müller cells with (+SC) or without (−SC) dietary supplementation. Gene ontology-enriched terms in dietary supplementation condition (+SC) was obtained thanks to the Database for Annotation, Visualization, and Integrated Discovery (DAVID). Red bars indicate the fold enrichment of the Gene Ontology (GO) and blue bars indicate the gene count. For each group, *n* = 6 mice.

To get an overall picture of the molecular modifications induced by the nutritional supplementation (+SC) on Müller cells, we performed a transcriptomic analysis after 4 days of treatment (Figure 1D and Figure 2). One hundred twenty-eight genes were deregulated (FoldChange>1.5) in +SC compared to −SC, with 72 upregulated and 56 downregulated (Supplemented data—Table S1). DAVID-based gene clustering chart analysis revealed an enrichment of several GO terms, mainly glutamatergic homeostasis related GO terms, in both molecular function (GO:0004364 Glutathione transferase activity, *p* = 0.0003) and biological process (GO:0006541 Glutamine metabolic process, *p* = 0.0778; GO:0006749 Glutathione metabolic process, *p* = 0.18) (Figure 2). Among these GO terms, *GLS1* (LogFC 0.77), *PHGDH* (LogFC 1.18), *GSTA1* (LogFC 0.90), *GSTA2* (LogFC 0.90), *GM10639* (LogFC 0.90) and *GM3776* (LogFC 0.90) genes were upregulated in +SC (Figure 2). In addition, we also observed deregulated genes related to glutamate metabolism such as *GOT2* (LogFC 0.74) but without enriched GO term. Therefore, these results suggested that nutritional supplement affects glutamatergic pathways.

**Figure 3:**
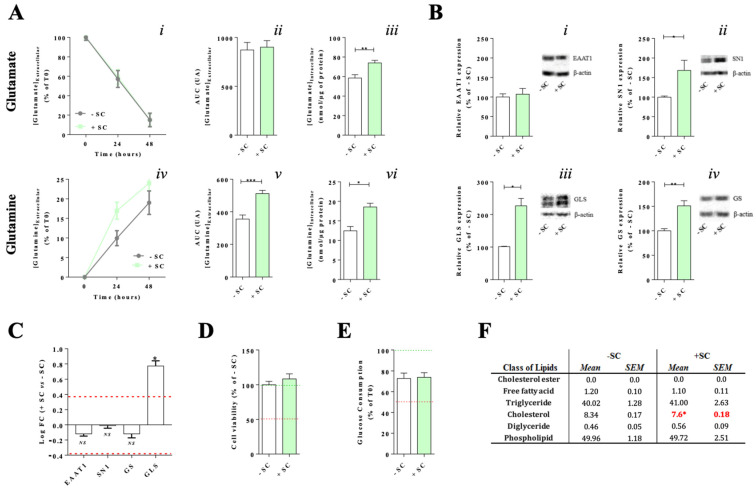
Glutamate/glutamine levels, related molecular partners expression, cells viability, glucose consumption and lipid composition in Müller cells with (+SC) or without (−SC) dietary supplementation. (**A**) Glutamate uptake (**i**,**ii**) and glutamine release (**iv**,**v**) are expressed respectively as concentration in extracellular medium and Area Under the Curve (AUC) over the 48 h of culture. Intracellular glutamate (**iii**) and glutamine (**vi**) concentrations were also determined after 48 h of culture. Glutamate/glutamine concentration were assessed by biochemical dosages and normalized on total protein amount per well. (**B**) Protein and (**C**) mRNA expressions of key molecular partners for the glutamate/glutamine cycle metabolism were investigated by Western blot and quantitative RT-PCR: (**i**) glutamate transporter (EAAT1), (**ii**) glutamine transporter (SN1), (**iii**) glutaminase (GLS) and (**iv**) glutamine synthetase (GS). Three independent experiments were performed. For each group, *n* = 10 mice. (**D**) Cell viability assessed by MTT, and (**E**) glucose consumption assessed by biochemical dosages, were evaluated at T48h. (**F**) Distribution of lipid classes in Müller cells at T0. For each group, *n* = 10 mice. All results are presented as mean ± SEM. Significant differences between groups are noted by * *p* < 0.05; ** *p* < 0.01; *** *p* < 0.001.

Based on these results, we focused our investigations on the Müller cells’ glutamate/glutamine metabolic cycle. Extracellular glutamate concentration decreased progressively by 85% between T0 and T48, independently from treatment [+SC or −SC] (Figure 3A(i)). The AUC was not significantly different between −SC [873 ± 76] and +SC [901 ± 66] (Figure 3A(ii)). In addition there was a similar amount of EAAT1 glutamate transporter protein and mRNA expressions [protein: 100 ± 8% for −SC vs. 124 ± 15% for +SC; mRNA: -0.12 ± 0.05 LogFC (+SC vs. −SC)] (Figure 3B(i),C) in both conditions. These results indicated that the supplement did not affect glutamate uptake. Surprisingly, intracellular glutamate level was significantly increased by 28% (*p* = 0.0016) in +SC compared to −SC [74 ± 3 nmol/µg of proteins vs. 58 ± 3 nmol/µg of proteins, respectively] (Figure 3A(iii)). This increase was associated to a significant transcriptional (*p* = 0.0256) and translational (*p* = 0.0108) up-regulation of the GLS glutaminase [protein: 227 ± 37% for +SC vs. 100 ± 2% for −SC; mRNA: +0.77 ± 0.12 LogFC (+SC vs. −SC)] (Figure 3B(iii) and C). These results indicated that supplement increased glutaminase and intracellular glutamate.

In +SC and −SC conditions, extracellular glutamine concentration increased progressively over 48 h (Figure 3A(iv)). However, glutamine release was significantly higher in +SC compared to −SC (*p* = 0.0001) as confirmed by AUC [512 ± 20 for +SC vs. 356 ± 24 for −SC] (Figure 3A(v)). This increase was associated with an increase (*p* = 0.0387) of SN1 translation without effect on its mRNA expressions [mRNA: −0.04 ± 0.06 LogFC (+SC vs. −SC); protein: 168 ± 25% for +SC vs. 100 ± 3% for −SC] (Figure 3B(ii),C). Surprisingly, intracellular glutamine was also significantly increased by 58% (*p* = 0.0101) in +SC compared to −SC [0.019 ± 0.0009 µmol/µg of proteins for +SC vs. 0.012 ± 0.001 µmol/µg of proteins for −SC] (Figure 3A(vi)). In our conditions, this increase was associated with a significant up-regulation of glutamine synthetase (GS) protein translation (*p* = 0.0011) without mRNA variation [protein: 150 ± 10% for +SC vs. 100 ± 4% for −SC; mRNA: −0.12 ± 0.09 LogFC (+SC vs. −SC)] (Figure 3B(iv),C). Therefore, these results suggested that supplement increase glutamine synthesis. Nevertheless, the genic and biochemical modifications of the glutamate/glutamine metabolic cycle in +SC had no impact on cell viability [72.74 ± 5.24% for −SC vs. 73.91 ± 4.35% for +SC, *p* = 0.999] (Figure 3D) or glucose consumption [100.00 ± 4.90% for −SC vs. 108.50 ± 7.38% for +SC, *p* = 0.969] (Figure 3E). Similarly, nutritional supplementation did not have any significant effect on lipid class composition except a slight modification of cholesterol levels [8.34 ± 0.17% of total lipids for −SC vs. 7.6 ± 0.17% of total lipids for +SC] (Figure 3F). No difference was observed in the fatty acid content of cells, and especially in the omega-3 polyunsaturated fatty acids (PUFAs) eicosapentaenoic acid (EPA) and docosahexaenoic acid (DHA) (data not shown).

All together, these results showed the nutritional supplement leaded to modifications of the glutamate/glutamine metabolic cycle in Müller cells, mainly characterized by an increase of glutamine synthesis independently from glutamate uptake.

### 3.2. Effects of Sub-Lethal Oxidative Stress on Müller Cells Glutamate/Glutamate Metabolic Cycle with or without Nutritional Supplementation

First, we investigated the effect of a H_2_O_2_ dose range concentration (from 0 up to 800 µM) on Müller cells viability (Figure 5B) and glucose consumption (Figure 5C). In the following experiments, we had chosen 100 µM and 200 µM conditions as sub-lethal oxidative stress since it did not affect any of both parameters.

**Figure 4:**
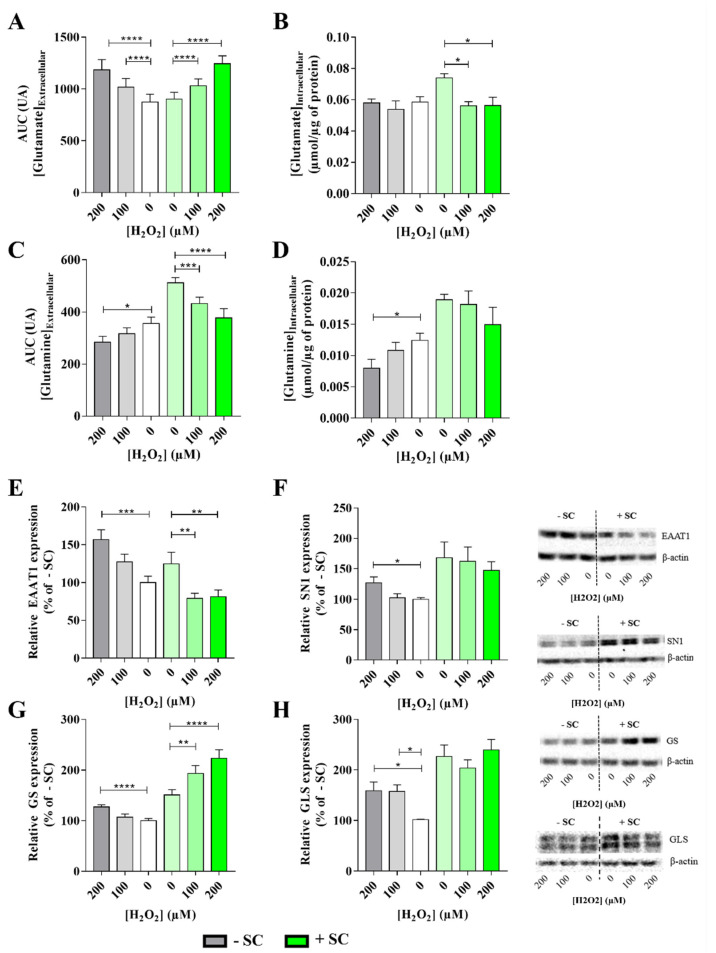
Sub-lethal oxidative stress in Müller cells with (+SC) or without (−SC) dietary supplementation. (**A**) Glutamate uptake and (**C**) glutamine releases were expressed as Area Under the Curve (AUC) over the 48 h of culture. Intracellular (**B**) glutamate and (**D**) glutamine concentrations after 48 h of culture were determined in presence of sub-lethal oxidative stress (100 and 200 µM H_2_O_2_) and with (+SC) or without (−SC) dietary supplementation. Glutamate and glutamine concentrations were assessed by biochemical dosages and normalized on total protein amount per well. For each group, *n* = 10 mice. Protein expression of key molecular partners for glutamate/glutamine cycle metabolism such as (**E**) glutamate transporter EAAT1, (**F**) glutamine transporter (SN1), (**G**) glutamine synthetase (GS) and (**H**) glutaminase (GLS) were investigated by Western blot determined in presence of sub-lethal oxidative stress (100 and 200 µM H_2_O_2_) and with (+SC) or without (−SC) dietary supplementation. Representative blots of results obtained for each protein assessed. Three independent experiments were performed with similar results. For each group, *n* = 6 mice. All results are presented as mean ± SEM. Significant differences between groups are noted by * *p* < 0.05; ** *p* < 0.01; *** *p* < 0.001; **** *p* < 0.0001.

Without supplementation (−SC), extracellular glutamate concentration decreased over time whatever the oxidative stress conditions (100 µM or 200 µM H_2_O_2_) (Data not shown). However, H_2_O_2_ reduced glutamate uptake in a dose-response manner (Figure 4A). Indeed, AUC was significantly higher by 15% for 100 µM H_2_O_2_ (1016 ± 84 AU) and by 35% for 200 µM H_2_O_2_ (1183 ± 100 AU) compared to 0 µM H_2_O_2_ (873 ± 76 AU) (*p* < 0.0001). Surprisingly, the glutamate internalizing protein expression, EAAT1 was increased by 27% for 100 µM H_2_O_2_ and significantly (*p* = 0.0008) increased by 56% for 200 µM (127 ± 10% for 100 µM H_2_O_2_, 156 ± 13% for 200 µM H_2_O_2_ and 100 ± 8% for untreated conditions) (Figure 4E). Intracellular glutamate was not affected by H_2_O_2_ whatever the concentration was (54 ± 5 nmol/µg of protein for 100 µM H_2_O_2_ and 58 ± 3 nmol/µg of protein for 200 µM H_2_O_2_ and 59 ± 3 nmol/µg of protein for untreated conditions) (Figure 4B). This stable level was associated with a significant (*p* = 0.0156) increase of GLS protein expression by 58% for both 100 and 200 µM H_2_O_2_ (158 ± 22% for 100 µM H_2_O_2_, 159 ± 28% for 200 µM H_2_O_2_ and 100 ± 2% for untreated conditions, respectively) suggesting a new synthesis of intracellular glutamate (Figure 4H).

Extracellular glutamine concentration increased over time in −SC cells exposed to 100 µM or 200 µM H_2_O_2_ (Data not shown). However, H_2_O_2_ reduced glutamine release in a dose-dependent manner as observed on the AUC decrease by 12% for 100 µM H_2_O_2_ and by 25% (*p* = 0.033) for 200 µM H_2_O_2_ (317 ± 22% for 100 µM H_2_O_2_, 284 ± 23% for 200 µM H_2_O_2_ vs. 356 ± 24% for 0 µM H_2_O_2_) (Figure 4C). Surprisingly, SN1 expression, the externalizing glutamine transporter protein, was significantly increased (*p* = 0.020) by 27% in 200 µM H_2_O_2_ (127 ± 10% vs. 100 ± 3% for 0 µM H_2_O_2_) (Figure 4F). Regarding intracellular glutamine, it was significantly (*p* = 0.032) decreased for 200 µM H_2_O_2_ (8 ± 1 nmol/µg of protein for vs. 12 ± 1 nmol/µg of protein for 0 µM H_2_O_2_) (Figure 4D) despite a significant (*p* < 0.0001) upregulation of GS protein expression (Figure 4G; 127 ± 5% for 200 µM H_2_O_2_ vs. 100 ± 4% 0 µM H_2_O_2_).

Therefore, in −SC, although sub-lethal oxidative stress reduced glutamate uptake, Müller cells maintained intracellular glutamate level at the expense of glutamine by increasing GLS over GS expression (Figure 4H).

With supplementation (+SC), glutamate concentration in culture medium decreased over time with or without sub-lethal (100 µM or 200 µM H_2_O_2_) conditions (Data not shown). However, H_2_O_2_ reduced glutamate uptake in a dose-dependent manner (Figure 4A) as shown by the extracellular glutamate AUC. Indeed, AUC was significantly increased (*p* < 0.0001) by 13% for 100 µM H_2_O_2_ (1031 ± 66 for 100 µM H_2_O_2_ vs. 902 ± 67 for untreated conditions) and by 38% (*p* < 0.0001) for 200 µM H_2_O_2_ (1244 ± 76 for 200 µM H_2_O_2_ vs. 902 ± 67 for untreated conditions) compared to 0 µM H_2_O_2_. This result is associated with a significant decrease of EAAT1 expression by 36% for 100 µM H_2_O_2_ (*p* = 0.0033) and by 35% for 200 µM H_2_O_2_ (*p* = 0.0065) (79 ± 7% for 100 µM H_2_O_2_, 81 ± 9% for 200 µM H_2_O_2_ and 125 ± 15% for 0 µM H_2_O_2_) (Figure 4E), and also associated with a significant decrease of intracellular glutamate concentration to a basal level whatever the H_2_O_2_ concentration was (56 ± 3 nmol/µg of protein for 100µM H_2_O_2_, *p* = 0.031; 56 ± 7 nmol/µg of protein for 200 µM H_2_O_2_ *p* = 0.033 and 74 ± 3 nmol/µg of protein for 0 µM H_2_O_2_) (Figure 4B). Interestingly, in presence of sub-lethal oxidative conditions (100 µM and 200 µM), the intracellular glutamate concentration is similar to the −SC ones but, surprisingly, whatever the oxidative stress was, the GLS expression stayed upregulated in +SC (204 ± 27% for 100 µM H_2_O_2_, 240 ± 34% for 200 µM H_2_O_2_ and 227 ± 37% for 0 µM H_2_O_2_) (Figure 4H).

In +SC, glutamine concentration in culture medium increased over time with or without sub-lethal (100 µM or 200 µM H_2_O_2_) conditions (Data not shown). However, H_2_O_2_ reduced glutamine release in a dose-dependent manner as observed by the AUC which were significantly decreased (*p* = 0.0002) by 15% for 100 µM H_2_O_2_ (433 ± 24 AU for 100 µM H_2_O_2_ vs. 512 ± 20 AU for 0 µM H_2_O_2_) and significantly (*p* < 0.0001) decreased by 26% for 200 µM H_2_O_2_ (377 ± 35 AU for 200 µM H_2_0_2_ vs. 512 ± 20 AU for 0 µM H_2_O_2_) (Figure 4C). However, intracellular glutamine concentration was not significantly modified by H_2_O_2_ treatments (18 ± 2 nmol/µg of protein for 100 µM H_2_O_2_, 15 ± 2 nmol/µg of protein for 200 µM H_2_O_2_ and 19 ± 1 nmol/µg of protein for 0 µM H_2_O_2_) (Figure 4D) as well as SN1 expression (162 ± 23% for 100 µM H_2_O_2_, 147 ± 14% for 200 µM H_2_O_2_ and 168 ± 26% for 0 µM H_2_O_2_) (Figure 4F). Nevertheless, a H_2_O_2_ dose-dependent response was observed for GS expression since it was significantly increased by 28% for 100 µM H_2_O_2_ (193 ± 15% for 100 µM H_2_O_2_ vs. 150 ± 10% for 0 µM H_2_O_2_; *p* = 0.008) and by 48% for 200 µM H_2_O_2_ (223 ± 16% for 200 µM H_2_O_2_ vs. 150 ± 10% for 0 µM H_2_O_2_; *p* < 0.0001) (Figure 4G). Therefore, the decrease in extracellular glutamine was not correlated neither to an increase of intracellular glutamine concentration nor to a decrease of SN1 expression despite an upregulation of GS protein.

These results suggest that in +SC, in oxidative condition, the level of intracellular glutamine is maintained by an increase of its synthesis by GS from a glutamate source other than the one added in the medium. Therefore, nutritional supplementation (+SC) seems to promote maintenance of a high level of glutamine in Müller cells by enhancing glutamine synthesis.

### 3.3. Effects of Nutritional Supplementation on Müller Cells Stress Markers

**Figure 5:**
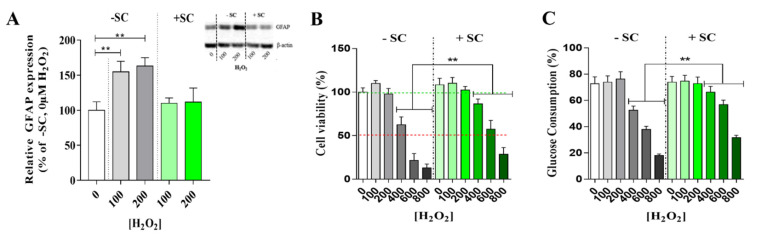
Müller cells reactivity and viability in oxidative stress conditions with (+SC) or without (−SC) dietary supplementation. (**A**) Müller cell reactivity was assessed by the Glial Fibrillary Acidic Protein (GFAP) expression in presence of sub-lethal oxidative stress (100 and 200 µM H_2_O_2_) and with (+SC) or without (−SC) dietary supplementation. GFAP expressions were assessed by Western blot. Representative blots of results obtained for each condition assessed. (**B**) Cell viability and (**C**) glucose consumption were evaluated after 48 h of cell culture with (+SC) or without (−SC) dietary supplementation and a ranging dose of H_2_O_2_. Cell viabilities are expressed in percentage of the result obtained in 0 µM H_2_O_2_. Glucose consumptions are expressed as the percentage of glucose of the initial amount (known at T0) which had been consumed. For each group, *n* = 6 mice. All results are presented as mean ± SEM. Significant differences between groups are noted by ** *p* < 0.01.

Müller cell reactivity was assessed by labelling its specific marker, Glial Fibrillary Acidic Protein (GFAP). As expected, H_2_O_2_ at 100 or 200 µM induced a significant (*p* < 0.01) 54% and 63%, respectively, increase of GFAP expression in (−SC) (Figure 5A). Interestingly, in (+SC), H_2_O_2_ at 100 or 200 µM had no effect on GFAP expression (Figure 5A). In addition, cell viability was significantly higher in (+SC) compared to (−SC) cells by 24% at 400 µM H_2_O_2_ (*p* = 0.0233) and by 35% at 600 µM H_2_O_2_ (*p* = 0.0012) (Figure 5B). Cell viability correlated with glucose consumption (Figure 5C).

Therefore, it appears that supplementation reduced Müller cells reactivity to oxidative stress thus preventing cell death in lethal oxidative stress conditions.

## 4. Discussion

Ocular supplements are routinely recommended for retinal degenerative diseases such as Age-related Macular Degeneration (AMD). However, little is known about their preventive mechanisms against retinal damage. Considering the importance of Müller cells in the retina, especially in photoreceptors cell neuroprotection [6], we raised the question of their involvement in the preventive effect of ocular supplementation. We particularly focused on the glutamate/glutamine metabolic cycle which is a major actor of Müller cell-associated neuroprotection [6] in both normal and oxidative stress conditions.

### 4.1. Dietary Supplementation Modifies Glutamate/Glutamine Metabolic Cycle in Far of Glutamine Synthesis

Important metabolic couplings exist among various cells through the use of common substrates and the exchange of several metabolic intermediates such as glutamate or glutamine [30,31]. In the retinal tripartite glutamatergic synapse, the fine control of glutamate level is ensured by Müller cells controlling the glutamate/glutamine metabolic cycle. The metabolic cycle aims to recapture glutamate from the synaptic cleft to maintain homeostasis, and to provide glutamine to replenish neurotransmitter pools in neurons, and thus avoid their excitotoxicity [6].

In this study, as expected, Müller cells were able to uptake glutamate, which could then be converted into glutamine by the glutamine synthetase (GS). Then, glutamine was released in culture medium, and acted as the driving force for glutamate uptake and GS activity to avoid any glutamate toxicity [32,33,34]. Interestingly, in high glutamate concentration [19] as used in this study to mimic glutamate toxicity involved in retinal degeneration [20,21], the main striking effect of dietary supplementation on Müller cells is the modulation of the glutamate/glutamine cycle in favor of glutamine release. The increase in GS, responsible for conversion of glutamate into glutamine [35], leads to an increase in intracellular glutamine as well as in glutamine release from Müller cells. This was correlated to an up-regulation of the membrane externalizing transporter SN1. The direct relationship between the glutamine synthesis, glutamine release and extracellular glutamine level is in accordance with the literature [36]. However, while glutamine increase is usually linked to an upregulation of glutamate internalizing transporter (EAAT1) [6,37,38] leading to an increase in glutamate uptake [39] and enhancing GS activity [34], we did not observe any effect of the supplementation on extracellular glutamate or EAAT1. Therefore, intracellular glutamate does not origin from an extracellular compartment, but from a regulation of intracellular metabolic pathway involving a new carbonate source for glutamate synthesis. Glutamate can be produced 1/from glucose through a metabolic pathway that begins with conversion of glucose to pyruvate which enter the Krebs cycle (TCA cycle) [40,41], 2/from protein catabolism producing amino acid free, such as glutamine leading to glutamate by deamidation [40,42]. Since we did not observe any change in glucose consumption with the supplementation it is unlikely for glutamate to originate from the Krebs cycle. On the other hand, the observed enrichment of GO term (GO:0032435) named “negative regulation of proteasomal ubiquitin-dependent protein catabolic process” (Figure 2) supports an up-regulation of protein breakdown. So, supplementation would enhance protein breakdown leading to an increase in glutamate which in turn would have led to an increased glutamine synthesis by GS. This would have been be balanced by an increase in GLS retroconverting glutamine to glutamate, in order to maintain stoichiometric amounts of glutamate and ammonium ions [43] and/or to the bioenergetics needs of Müller cells [44,45].

### 4.2. Synergic Effect of the Nutritional Supplementation Compounds on Glutamine Enhancement

A seven day supplementation was shown to induce changes in lipid composition such as DHA, EPA and AA in vivo [16] in rats as well as in vitro in retinal pigment epithelial cells (ARPE19) (personal communication, Ramchani-Ben Othman, K). However, in the present study, we did not notice any significant modification in Müller cells’ lipid composition, and especially their fatty acid content, after 4 days of supplementation. Therefore, the observed impact of our short-term exposure on the glutamate/glutamine metabolic cycle is independent from cells’ lipid composition, in opposition to a long-term exposure to PUFAs [46,47,48]. However, the participation of DHA from supplements to the reduction of glutamate uptake without modifications in the expression of membrane-associated astroglial glutamate transporters (EAAT1 and GLT-1) [49] but with a decreased GS expression [48] cannot be excluded. Therefore, the supplement could have a direct cellular signaling effect through the PUFAs it contains.

Among the other compounds, resveratrol can significantly interfere with the glutamate/glutamine cycle. It has been shown that short-term resveratrol exposure from one to 100 µM induced a linear increase in glutamate uptake and glutamine synthase activity [50]. In addition, in primary cortical astrocytes, 25 µM resveratrol increased glutamate uptake and glutamine content, whereas at 250 µM resveratrol decreased glutamate uptake but increase GS activity [50]. Similarly, resveratrol improved the glutamate metabolism in Müller cells via up-regulation of GS protein expression and activity in diabetic retinas, providing protective effect against retinal degeneration [51]. Even if our experimental conditions are quite different in terms of cellular model (Müller cell), concentration used (113 µM), exposure timing and nutritional supplementation type (complex formulation), we could nevertheless suggest a participation of resveratrol on the glutamate/glutamine cycle modulation. More recently, it was shown that resveratrol administration enhances the activity of the ubiquitin-proteasome system [52] and promotes proteasomal degradation [53]. This mechanism could explain the modification of proteasomal ubiquitin-dependent GO term observed in our model, suggesting that resveratrol is involved in providing carbonate source for the new glutamate synthesis.

Resveratrol and PUFAs are the major components of the supplementation mixture, and the literature has brought evidence of several effects of these substances on glutamatergic homeostasis modulation. Consequently, even if the dietary supplement contains other compounds, we could hypothesize that the observed effect on the glutamate/glutamine metabolic cycle in our experimental conditions might originate mainly from the synergic effect of both PUFAs and resveratrol.

### 4.3. Dietary Supplementation Induced a Glutamine Buffering to Delay Oxidative Stress Impact: High Glutamine Level as a Key Target for Retinal Neuroprotection

Glutamate is also important for maintaining levels of antioxidant glutathione GSH [54]. Transcriptomic analysis demonstrates that nutritional supplementation impacts glutamate homeostasis GO terms as discussed above, but also classical pathways for oxidative stress defense. Indeed, “GO:0004364 Glutathione transferase activity” and “GO:0006749 Glutathione metabolic process” were enriched, and the associated genes such as GSTA1, GSTA2, GM10639 and GM3776 were upregulated. Based on these observations, we raised the question about the effect of oxidative stress on the glutamate/glutamine metabolic cycle of Müller cells and the protective effect of nutritional supplementation against this oxidative stress. Herein, we have chosen sub-lethal oxidative stress conditions to investigate the early oxidative stress response.

**Figure 6:**
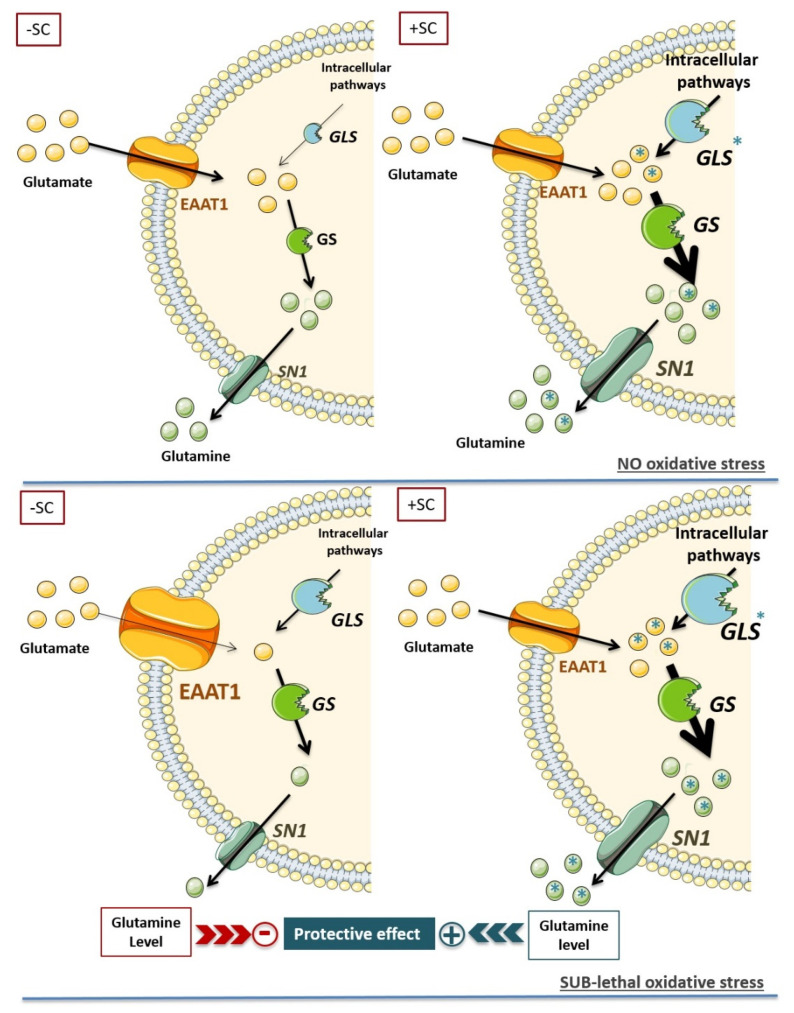
Schematic representation of nutritional supplementation impact on Müller cells’ glutamine homeostasis with or without oxidative stress. The upper panels represent the glutamate/glutamine cycle without (−SC) and with (+SC) nutritional supplementation in absence of oxidative stress. The lower panels present the glutamate/glutamine cycle without (−SC) and with (+SC) nutritional supplementation in presence of oxidative stress. EAAT1: glutamate transporter; SN1: glutamine transporter; GLS: glutaminase; GS: glutamine synthetase. The asterisks (*) represent the new synthesis of glutamate or glutamine induced by action of SC on intracellular pathways. The size of the characters or arrows in the figure’s images represent the significance of the process that they illustrate or the quantity of a protein or enzyme: the bigger the size, the more significant the biological item is.

As previously described in several different experimental models [55,56,57,58], we showed that oxidative stress affects the glutamate/glutamine metabolic cycle of the Müller cells by decreasing the glutamate uptake, the glutamine synthesis, and thus the glutamine release. Supplemented Müller cells exposed to oxidative stress had a glutamate/glutamine metabolic cycle reaching the level of untreated cells. This is particularly interesting for glutamine levels, which are essential for neuron function. Therefore, we hypothesize that the high glutamine level induced by nutritional supplementation protects Müller cells by creating a buffering against oxidative stress impact. To go further, increasing the oxidative stress up to 800 µM H_2_O_2_ clearly showed an offset of cell death in the supplemented conditions compared to the unsupplemented ones. Consequently, by increasing the level of glutamine, the nutritional compound protects Müller cells against oxidative mechanism. Therefore, we can assume that in vivo, the supplement offered retinal protection against oxidative stress induced by light [16] partly by acting on glutamine level. Our data and conclusions are in accordance with literature, since impairments of glutamine level associated to GS defect are clearly highlighted in many diseases leading to neuronal glutamate depletion [59]. Indeed, in photoreceptor cells death (related to inherited photoreceptor degeneration, retinal light injury, or retinal detachment), Müller cells display a drop in their GS expression and glutamine level [60,61,62]. Similarly, a decline in GS expression and activity was also observed under ischemic, inflammatory, and traumatic conditions or in glaucoma [7,63,64,65]. These observations are in accordance with experimental data using pharmacological inhibitor of the GS activity in the retina. In substance, in vivo retinal inhibition of GS led to a loss of free glutamate content in bipolar and ganglion cells, and thus to rapid blindness in animals [8,66]. Moreover, downregulation of GS using siRNA results in the breakdown of the blood-retinal barrier [67], suggesting that glutamate homeostasis defect is also associated to the integrity of the blood-retinal barrier. Consequently, we could raise the hypothesis that the control of glutamine level, especially its high concentration, is a key point for neuronal retinal cells’ protection, and thus the photoreceptor cells’ survival (Figure 6).

In conclusion, our data demonstrate that enrichment of Müller cell environment with omega-3, resveratrol and minerals contained in a dietary supplement offers a preventive effect against oxidative stress by promoting glutamine synthesis. This increase of intracellular glutamine synthesis results from intracellular metabolic pathway deviation and probably protein breakdown enhancement. Such Müller cell mechanisms and their consequences on the entire retina could explain the in vivo protective effect of dietary supplement in light-induced damage [16] and its beneficial effect in human patients affected by Age-related Macular Degeneration (AMD) [68]. Further studies will be conducted in order to evaluate the effect of sub-lethal oxidative stress on the glutamate/glutamine cycle in Müller cells cultured with lower or no glutamate in media and to quantify the variations of glutamate/glutamine cycles in retina from in vivo supplemented animals subjected to oxidative stress induced by light exposure.

## Data Availability

The original contributions presented in the study are included in the article and Supplementary Materials. Further inquiries can be directed to the corresponding authors.

## Supplementary Materials

The following are available online at https://www.mdpi.com/article/10.3390/nu13093216/s1; Table S1: Deregulated genes in Müller cells with dietary supplementation.
